# Effects of incubation with endocannabinoids on the expression of endocannabinoid and inflammatory components following an ex vivo lipopolysaccharide challenge in leukocytes of dairy cows

**DOI:** 10.3168/jdsc.2025-0866

**Published:** 2025-12-13

**Authors:** P. dos Santos Silva, Y. Butenko, L. Hubner, B. Shattenstein, M. Zachut

**Affiliations:** 1 Department of Ruminant Science, Institute of Animal Sciences, Agricultural Research Organization, Volcani Institute, Rishon LeZion, Israel, 7505101; 2 Department of Animal Science, The Robert H. Smith Faculty of Agriculture, Food and Environment, The Hebrew University of Jerusalem, Rehovot, Israel, 7610001

## Abstract

The endocannabinoid system (ECS) is involved in regulating immune functions in leukocytes. An inflammatory stimulus, specifically LPS, can modulate the activity of endocannabinoids (eCB) receptors, and eCB within the leukocytes can further exert either pro- or anti-inflammatory effects on the immune function of these cells. The effects of exogenous eCB on the cellular inflammatory responses of bovine leukocytes are largely unexplored; therefore, we aimed to evaluate the effects of incubation with the main eCB *N*-arachidonoylethanolamide (AEA/anandamide) or 2-arachidonyglycerol (2-AG) on gene expression of eCB and inflammatory mediators following an ex vivo LPS challenge in dairy cow leukocytes. To this end, whole blood from mid-lactation dairy cows (n = 6) were subjected to ex vivo incubation with eCB (control [CTL], AEA at 0.29 µ*M*, or 2-AG at 0.26 µ*M*) for 2 h, followed by stimulation with or without LPS (10 ng/mL) for an additional 2 h. Overall, there were 6 treatments for cells from each cow: CTL, AEA, and 2-AG without LPS stimulation, and CTL+LPS, AEA+LPS, and 2-AG+LPS. Subsequently, RNA was extracted from leukocytes and assayed for gene expression levels via real-time quantitative PCR. First, we examined the main effects of LPS stimulation across eCB treatments: LPS decreased expression of the eCB receptors cannabinoid receptor 2 (*CNR2*), G protein-coupled receptor 55 (*GPR55*), and peroxisome proliferator-activated receptor gamma (*PPARG*). Furthermore, LPS increased expression of the eCB enzymes N-acyl phosphatidylethanolamine phospholipase D (*NAPEPLD*) and monoglyceride lipase (*MGLL*) while reducing the expression of diacylglycerol lipase B (*DAGLB*) compared with non-LPS-stimulated groups. Then, we examined the main effects of eCB treatments on gene expression: incubation with AEA increased expression of cannabinoid receptor 1 (*CNR1*) in leukocytes, whereas 2-AG increased the expression of the *CNR2* and tended to increase *GPR55*. In addition, 2-AG increased the expression of fatty acid amide hydrolase (*FAAH*) and tended to increase the expression of *NAPEPLD*. As expected, LPS increased the expression of inflammatory markers; however, incubation with eCB had no discernible effects on these genes. Taken together, ex vivo exposure of dairy cow leukocytes to AEA or 2-AG, with or without stimulation with LPS, resulted in differential effects on the expression of eCB receptors and enzymes, but we did not detect effects of exogenous eCB on the expression of inflammatory genes following an LPS challenge. The findings of the present study provide the first reductionist step in understanding the relationship between the ECS and inflammatory responses in immune cells of dairy cows. The complexity of the regulation of immune function in leukocytes, and its potential interplay with eCB, requires further studies to comprehensively elucidate the cellular mechanisms underlying these responses.

## Highlights

• LPS alters endocannabinoid system gene expression in bovine leukocytes.
• Incubation with endocannabinoids had minor effects on leukocyte inflammatory genes.
• 2-AG upregulated expression of fatty acid amide hydrolase in leukocytes.

## Graphical Abstract

**Figure:**
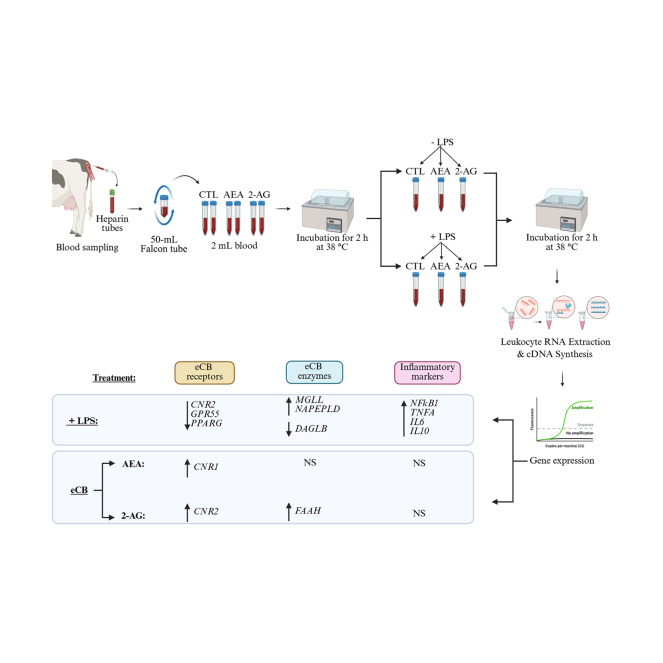
**Summary:** In this ex vivo study, dairy cow whole blood samples were incubated with the endocannabinoids N-arachidonoylethanolamide (AEA) or 2-arachidonyglycerol (2-AG) at ~0.3 μM for 2 hours and then stimulated or not with lipopolysaccharide (LPS) for 2 hours. After RNA extraction from leukocytes, gene expression was analyzed by PCR. The LPS challenge reduced the relative expression levels of *CNR2*, *GPR55*, *PPARG*, and *DAGLB*, but increased expression of *MGLL* and *NAPEPLD*, as well as the inflammatory markers *NFkB1*, *TNFA*, *IL6*, and *IL10*. Incubation with 2-AG increased expression of *CNR2* and *FAAH* compared with control, whereas AEA increased expression of *CNR1*. Incubation with AEA or 2-AG did not affect the expression of inflammatory genes compared with control. The complexity of crosstalk between immune regulation and endocannabinoid system activity in leukocytes requires further investigation to fully elucidate the mechanistic foundations of these responses. Created in BioRender.com.

The endocannabinoid system (**ECS**) participates in immune regulation in mammals, mediated mainly through the activity of the cannabinoid receptor 2 (CB2/*CNR2*), which is predominantly expressed in immune cells (Rakotoarivelo et al., 2024). The endocannabinoid (**eCB**) ligands are lipid derivates of fatty acids, synthesized on demand in most mammalian cell types (Silvestri and Di Marzo, 2013; Zachut et al., 2022). The 2 primary and best-characterized eCB are *N*-arachidonoylethanolamide (**AEA**, or anandamide) and 2-arachidonyglycerol (**2-AG**), which engage the canonical membranal cannabinoid receptors 1 (CB1/*CNR1*) and *CNR2*, as well as orphan eCB receptors such as the G protein-coupled receptor 55 (GPR55), and nonclassical receptors belonging to the intracellular peroxisome proliferator-activated receptor (PPAR) family (Silvestri and Di Marzo, 2013; Zachut et al., 2022). The regulation of immune function by the ECS is extremely complex, and various experimental models have demonstrated both pro- and anti-inflammatory effects of ECS activation on immune responses (Zachut et al., 2025). Increasing levels of endogenous AEA and 2-AG by selective inhibition or deletion of their respective catabolizing enzymes fatty acid amide hydrolase (FAAH) and monoglyceride lipase (MGLL) are associated with reduced inflammation (Rakotoarivelo et al., 2024). Furthermore, 2-AG, which has a high affinity for *CNR2*, promotes its activation (Rakotoarivelo et al., 2024), which in turn has been shown to affect migration, proliferation, and cell death in immune cells, as well as secretion of cytokines, predominantly exerting anti-inflammatory and immunosuppressive effects (Basu and Dittel, 2011; Myers et al., 2021). Meanwhile, AEA and 2-AG and their metabolites can be metabolized by cyclooxygenase 2 into prostaglandins that promote proinflammatory responses (Rouzer and Marnett, 2011; Myers et al., 2021). Thus, the availability of AEA and 2-AG in leukocytes may regulate the immune response (Malek et al., 2015; Szafran et al., 2015). Adding another layer to the complex relationship between ECS and immunoregulation, inflammatory stimulus with LPS can also modulate the cellular activity of eCB receptors in immune cells, as it has been demonstrated that LPS lowers the expression of *CNR2* in RAW264.7 macrophages (Carlisle et al., 2002). Recently, we proposed that modulating the ECS, and specifically *CNR2* activation, could affect immune regulation in dairy cows (Zachut et al., 2025); however, information on the involvement of the ECS in immune function in ruminants is scarce. We hypothesized that exogenous AEA and 2-AG could have anti-inflammatory effects, and that LPS would affect the expression of *CNR2* and possibly other ECS components in the leukocytes. The objective of this ex vivo study was to evaluate the effects of AEA and 2-AG, with or without an LPS challenge, on the gene expression of ECS and inflammatory components in leukocytes of dairy cows.

The experimental protocol for the study was approved by the Volcani Center Animal Care Committee (approval number 997/23IL) and was performed in accordance with the relevant guidelines and regulations. The study was conducted at the Volcani Institute experimental farm in Rishon LeZion, Israel, during the winter season, and included 6 healthy multiparous Holstein dairy cows (average lactation number 2 ± 1, DIM 186 ± 3). The cows were group-housed in a shaded loose pen and fed a lactating cows' diet balanced for energy, protein, and ether extract content. For each cow, blood was collected from the coccygeal vein into 3 vacuum tubes containing lithium heparin (no. BD367526, Becton Dickinson Systems, Cowley, UK), transported to the laboratory within 10 min at room temperature, and blood from each cow was pooled into a single 50-mL Falcon tube. Aliquots of 2 mL from each cow were immediately transferred to 15-mL Falcon tubes and treated in quadruplicate as follows: (1) the control (**CTL**) group received 10 µL of ethanol absolute; (2) the AEA group received 10 µL of AEA at 20 µg/mL (0.29 µ*M*, dissolved in ethanol); (3) the 2-AG group received 10 µL of 2-AG at 20 µg/mL (0.26 µ*M*, dissolved in ethanol). The tubes were then incubated in a water bath at 38°C for 2 h. Next, 2 tubes from each treatment group received 20 µL of LPS at 1 µg/mL (in PBS), and the other 2 tubes in each group received 20 µL of PBS. Overall, blood from each cow was divided into 6 treatments: 3 treatments without LPS stimulation (CTL, AEA, and 2-AG) and 3 treatments with LPS stimulation (**CTL+LPS**, **AEA+LPS**, and **2-AG+LPS**), and all treatments were incubated further in a water bath at 38°C for an additional 2 h. The incubation times in this study were selected following a series of preliminary experiments, in which we examined different eCB incubation times (n = 6 cows in each experiment): in one experiment, leukocytes were simultaneously incubated with eCB and LPS for 2 h; and in the other, leukocytes were pre-incubated with eCB for 15 min, followed by 2 h of LPS challenge. However, in both preliminary experiments, only very minor or no eCB effects were observed on ECS and inflammatory genes. Previously, Walker et al. (2022) incubated bovine endothelial cells with LPS and AEA and showed that the greatest change in results occurred at an incubation time of 4 h post-AEA. Thus, we decided on the current setting (pre-incubation with eCB for 2 h, and 2 h with LPS). Cell stimulation was stopped by placing the tubes on ice, followed by centrifugation at 4°C for 10 min at 1,500 × *g*, followed by discarding of the supernatant. The samples were prepared for RNA purification, and the leukocyte pellets from duplicate tubes of each treatment were combined for RNA extraction with a Norgen Leukocytes RNA Purification Kit (no. 21200, Norgen BioTek Corp., Ontario, Canada). RNA yield and purity were evaluated using NanoDrop One (NanoDrop One Microvolume UV-Vis Spectrophotometer, Thermo Scientific, Shoham, Israel). First-strand cDNA was generated using the RevertAid First Strand cDNA Synthesis Kit (no. K1622, Thermo Fisher Scientific, Vilnius, Lithuania). Real-time quantitative PCR was conducted using a CFX Duet instrument (CFX Duet Real-Time PCR System, Bio-Rad Laboratories, Rishon LeZion, Israel) with the SYBR Green PowerTrack Master Mix (no. A46109, Applied BioSystems, Woburn, MA) and analyzed using Bio-Rad CFX Maestro Software version 2.3 (CFX Duet Real-Time PCR System, Bio-Rad Laboratories, Rishon LeZion, Israel).

The samples were examined for transcription levels of eCB receptors *CNR1*, *CNR2*, *GPR55*, and PPAR gamma (*PPARG*); eCB enzymes diacylglycerol lipase B (*DAGLB*), GATTCTGAGCCAAGCGTTC (forward), 5′-GGAGTATTCATAAAGGGATTTGCTG-3′ (reverse), N-acyl phosphatidylethanolamine phospholipase (*NAPEPLD*), *FAAH*, and *MGLL*; inflammatory components inflammatory components nuclear factor kappa B subunit 1 (*NFkB1*), 5′-GCAATCATCCACCTTCATAATCAG-3′ (forward), 5′-GCAAGTCCTCCACCACAG-3′ (reverse), tumor necrosis factor-α (TNF/*TNFA*), 5′-CTCTGGTTCAAACACTCAGGTC-3′ (forward), 5′-CGGAGAGTTGATGTCGGCTAC-3′ (reverse), interleukin 6 (*IL6*), 5′-CTGCTGGTCTTCTGGAGTATC-3′ (forward), 5′-TGTGGCTGGAGTGGTTATTAG-3′ (reverse), and interleukin 10 (*IL10*), 5′-CGCTGTCATCGCTTTCTG-3′ (forward), 5′-GCATCTTCGTTGTCATGTAGG-3′ (reverse). The rest of the primer sequences were previously described in dos Santos Silva et al. (2025). Relative gene expressions were calculated according to Bustin et al. (2025). Housekeeping genes peptidylprolyl isomerase A (*PPIA*), 5′-CAACCCCACCGTGTTCTTC-3′ (forward), 5′-GACTTGCCACCAGTACCATTA-3′ (reverse); ribosomal protein S9 (*RPS9*), 5′-CTGAAGCTGATCGGCGAGTA-3′ (forward), 5′-GCGGGTCTTTCTCATCCA-3′ (reverse); and ribosomal protein L4 (*RPL4*), 5′-AGGAGGCTGTGTTGCTTCTG-3′ (forward), 5′-CGCTGAGAGGCATAGACCTT-3′ (reverse) were assessed as candidate reference genes, and NormFinder version 21 software (https://www.moma.dk/software/normfinder) identified the combination of *RPS9* and *PPIA* as the most stable across this study.

The relative mRNA abundances of leukocytes (from 6 cows, each in 6 treatments) were analyzed using Proc Mixed (SAS version 9.4; SAS Institute Inc.), using the following model:

Y_ijk_ = μ + T_i_ + L_j_ + (T × L)_ij_ + A_k_ + ε_ijk_,

where Y_ijk_ = response observed for animal k under the treatment combination of eCB i (CTL, AEA, or 2-AG) and LPS challenge j (with or without LPS); μ = overall mean; T_i_ = fixed effect of the eCB treatment; L_j_ = fixed effect of the LPS challenge; (T × L)_ij_ = fixed interaction effect between eCB and LPS; A_k_ = random effect of the animal; and ε_ijk_ is the residual error. The data were analyzed using SAS 9.4 software. The model assumptions of normality and equality of variances were tested by the Shapiro-Wilk test on the model residuals and the *F*-test, respectively. Relative expression data are presented as LSM ± SEM, and differences were considered statistically significant at *P* ≤ 0.05, with tendency noted at 0.05 < *P* ≤ 0.10 by Tukey's test. Gene expression was analyzed for the main treatment effects of LPS, eCB, and their interaction, as well as individual comparisons between the 6 treatments.

The relative expressions of the eCB and inflammatory genes are presented in Table 1. First, we examined the main effect of LPS on gene expressions across eCB treatments: stimulation with LPS reduced the expressions of *CNR2* (*P_LPS_* < 0.0001), *GPR55* (*P_LPS_* < 0.0001), and *PPARG* (*P_LPS_* < 0.0001) compared with cells that were not stimulated with LPS, but no difference was observed in *CNR1* expression (*P_LPS_* = 0.16; Figure 1 A–D). Then, we examined the main effect of eCB on gene expressions across LPS stimulation: incubating leukocytes with eCB had significant but differential effects on the expression patterns of *CNR1* (*P_eCB_* = 0.03) and *CNR2* (*P_eCB_* = 0.02). Pooled analysis across LPS stimulation groups revealed increased *CNR1* expression in AEA treatment compared with 2-AG-treated leukocytes (*P* = 0.03), but results were similar to CTL (*P* = 0.87). In contrast, *CNR2* expression was significantly increased by 2-AG compared with CTL (*P* = 0.02); however, AEA treatment had no effect (*P* = 0.76; Figure 2 A, B). In addition, eCB tended to affect the expression of GPR55 (*P_eCB_* = 0.08), where 2-AG tended to increase the expression of *GPR55* compared with CTL (*P* = 0.06) but not compared with AEA (*P* = 0.34; Figure 2 C). No significant eCB effect was observed for *PPARG* expression (*P_eCB_* = 0.48; Figure 2 D). When analyzing the interaction eCB × LPS (Table 1), we found that LPS reduced the expressions of *CNR2* (*P_eCB_*_×_*_LPS_* = 0.01) in CTL+LPS compared with CTL (*P* = 0.0004), AEA (*P* = 0.003), 2-AG (*P* < 0.0001), AEA+LPS (*P* = 0.009), and 2-AG+LPS (*P* = 0.03). A reduction was also observed in *GPR55* gene expression (*P_eCB_*_×_*_LPS_* = 0.02) in the LPS-stimulated cells (CTL+LPS, AEA+LPS, and 2-AG+LPS) compared with non-LPS-stimulated cells (CTL, AEA, and 2-AG). However, the interaction eCB × LPS did not affect the expressions of *CNR1* and *PPARG* (Table 1).

**Table 1:** Relative expressions of eCB and inflammatory genes in dairy cow leukocytes, incubated ex vivo with AEA or 2-AG and then stimulated (+) or not (−) with LPS^1^

LPS	Treatment						SEM	*P*-value		
	CTL		AEA		2AG			eCB	LPS	eCB × LPS
	−	+	−	+	−	+				
eCB receptor										
*CNR1*	1.00	0.69	1.29	0.65	0.26	0.34	0.257	0.03	0.16	0.36
*CNR2*	0.99^a^	0.46^b^	0.91^a^	0.86^a^	1.06^a^	0.81^a^	0.081	0.02	<0.0001	0.01
*GPR55*	1.01^a^	0.45^b^	0.91^a^	0.65^b^	1.11^a^	0.60^b^	0.060	0.08	<0.0001	0.02
*PPARG*	1.00	0.23	0.99	0.29	1.07	0.30	0.057	0.48	<0.0001	0.75
eCB enzyme										
*NAPEPLD*	1.00^ab^	0.99^ab^	0.91^b^	1.25^a^	1.06^ab^	1.25^a^	0.077	0.08	0.004	0.05
*DAGLB*	1.00	0.72	0.91	0.67	1.03	0.76	0.076	0.34	<0.0001	0.97
*FAAH*	1.00	0.92	1.03	1.11	1.33	1.05	0.110	0.04	0.37	0.29
*MGLL*	1.00	3.70	0.97	3.88	0.88	3.36	0.398	0.58	<0.0001	0.78
Inflammatory marker										
*NFkB1*	1.00	4.32	0.98	4.38	1.05	4.08	0.343	0.58	<0.0001	0.79
*TNFA*	1.00	9.52	1.10	8.13	0.94	7.63	0.908	0.53	<0.0001	0.54
*IL6*	1.00	26.10	1.65	26.49	1.07	26.86	4.683	0.99	<0.0001	0.99
*IL10*	1.00	38.16	0.94	39.32	0.88	38.48	4.079	0.99	<0.0001	0.99

a,b Within a row, means with different superscripts differ at *P* ≤ 0.05 for effect of interaction LPS × eCB by Tukey's test.

1 Sample size is n = 6 cows in each treatment group.

**Figure 1:**
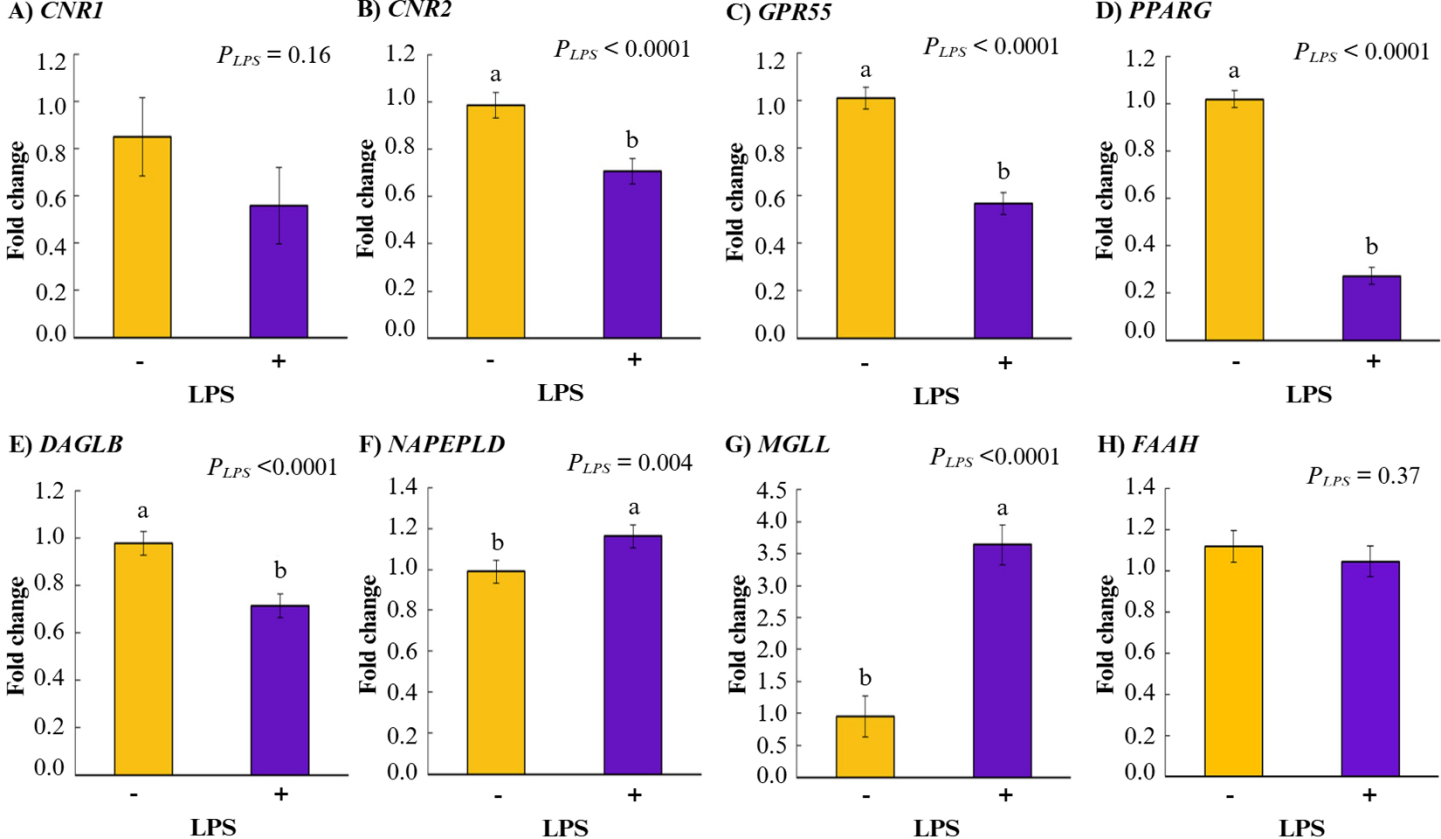
Main effects of LPS (*P_LPS_*) on gene expression of eCB receptors and enzymes in leukocytes of dairy cows. Effects of LPS stimulation (with [+] or without [−]) in dairy cow (n = 6) leukocytes on the relative gene expressions of (A) cannabinoid receptor 1 (*CNR1*), (B) cannabinoid receptor 2 (*CNR2*), (C) G protein-coupled receptor 55 (*GPR55*), and (D) peroxisome proliferator-activated receptor gamma (*PPARG*); (E) endocannabinoid (eCB) synthesizing enzymes diacylglycerol lipase β (*DAGLB*) and (F) N-acyl phosphatidylethanolamine phospholipase D (*NAPEPLD*); and eCB degrading enzymes (G) monoglyceride lipase (*MGLL*) and (H) fatty acid amide hydrolase (*FAAH*). Data show mean ± SEM; groups were pooled across eCB treatments. Means with different lowercase letters (a,b) differ at *P* ≤ 0.05 by Tukey's test. Created in BioRender.com.

**Figure 2:**
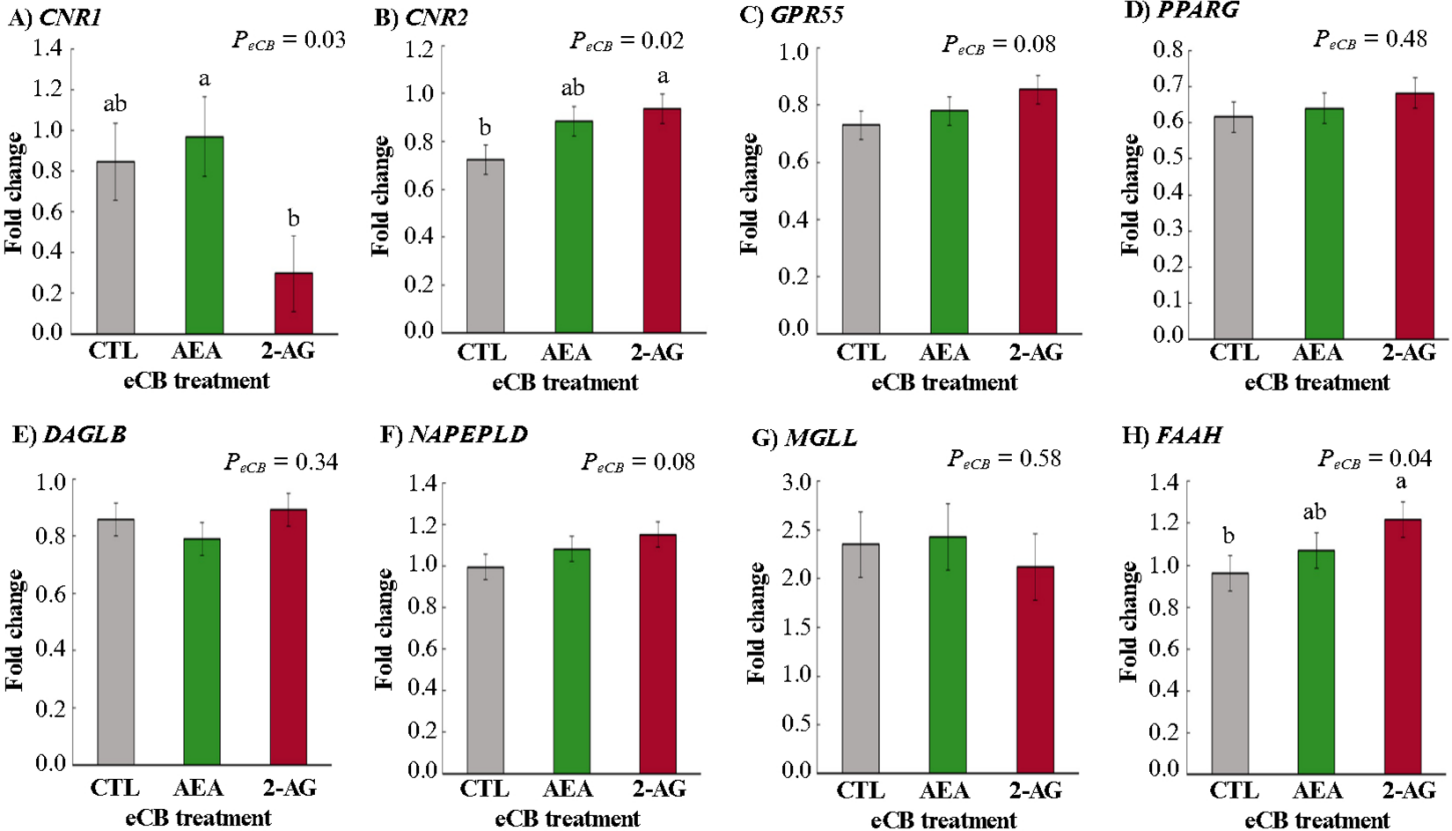
Main effects of exogenous AEA or 2-AG on the gene expressions of endocannabinoid (eCB) receptors and enzymes in leukocytes of dairy cows. Effects of eCB on relative gene expression of (A) cannabinoid receptor 1 (*CNR1*), (B) cannabinoid receptor 2 (*CNR2*), (C) G protein-coupled receptor 55 (*GPR55*), and (D) peroxisome proliferator-activated receptor gamma (*PPARG*); (E) synthetized enzymes diacylglycerol lipase β (*DAGLB*) and (F) N-acyl phosphatidylethanolamine phospholipase D (*NAPEPLD*); and degradation enzymes (G) monoglyceride lipase (*MGLL*) and (H) fatty acid amide hydrolase (*FAAH*) stimulated with LPS in dairy cow (n = 6) leukocytes. 2-AG = 2-arachidonoylglycerol; AEA = N-arachidonoylethanolamide; CTL = control. Data show mean ± SEM; groups were pooled across LPS treatment. Means with different lowercase letters (a,b) differ at *P* ≤ 0.05 by Tukey's test. Created in BioRender.com.

We evaluated the effects of LPS and eCB on expression levels of the ECS key metabolic enzymes *NAPEPLD* and *FAAH*, which synthesize and degrade AEA, respectively; as well as in their counterparts in 2-AG metabolism, *DAGLB* and *MGLL*. Pooled analysis across eCB treatment groups indicated an opposing effect of LPS on these 2 enzyme-pairs (Figure 1 E–H): *DAGLB* expression was inhibited by LPS (*P_LPS_* < 0.0001), whereas *MGLL* was enhanced (*P_LPS_* < 0.0001). In contrast, LPS stimulation increased the relative gene expressions of *NAPEPLD* (*P_LPS_* = 0.004) but did not significantly affect the expression of *FAAH* (*P* = 0.37) compared with non-LPS-stimulated cells. Pooled analysis across LPS stimulation groups showed that 2-AG had a stimulatory effect on expression of *FAAH* (*P* = 0.03; Figure 2 H) and tended to increase *NAPEPLD* (*P* = 0.06; Figure 2 F) compared with CTL, but we did not detect differences in the expression of eCB enzymes in AEA-treated cells compared with CTL (*FAAH*: *P* = 0.29; *NAPEPLD*: *P* = 0.54; Figure 2 H and F). No significant individual effects of eCB were detected for gene expressions of *DAGLB* (*P*_eCB_ = 0.34, Figure 2 E) or *MGLL* (*P_eCB_* = 0.58; Figure 2 G). The interaction eCB × LPS showed that LPS+eCB increased the expression of *NAPEPLD* (*P_eCB_*_×_*_LPS_* = 0.05) in AEA+LPS (*P* = 0.02) and 2-AG+LPS (*P* = 0.02) compared with AEA. However, they were similar to CTL+LPS, CTL, and 2-AG. The interaction did not affect the expression of any other enzymes analyzed (Table 1).

As expected, pooled analysis across eCB groups indicated that LPS increased the expression of inflammatory genes; it increased the gene expression of *NFkB1* (4.26 vs. 1.01 fold change [**FC**] in LPS and CTL, respectively, SEM = 0.256; *P_LPS_* < 0.0001), *TNFA* (8.43 vs. 1.01 FC in LPS and CTL, respectively, SEM = 0.562; *P_LPS_* < 0.0001), *IL6* (26.49 vs. 1.24 FC in LPS and CTL, respectively, SEM = 3.249; *P_LPS_* < 0.0001), and *IL10* (38.65 vs. 0.94 FC in LPS and CTL, respectively, SEM = 2.803; *P_LPS_* < 0.0001) compared with CTL. In contrast, eCB did not affect the expression of any of the examined genes *NFkB1* (*P_eCB_* = 0.58), *TNFA* (*P_eCB_* = 0.53), *IL6* (*P_eCB_* = 0.99), and *IL10* (*P_eCB_* = 0.99); and eCB × LPS interactions were not significant (Table 1).

In this study, we examined the effects of ex vivo incubation with AEA or 2-AG, followed by an inflammatory LPS challenge, on expression of ECS and inflammatory genes, as the first reductionist step to explore this intricate regulation in leukocytes of dairy cows. We found that LPS downregulated gene expression of the eCB receptors (*CNR2*, *GPR55*, and *PPARG*), increased expressions of the eCB enzymes *NAPEPLD* and *MGLL*, and decreased *DAGLB* compared with controls in bovine leukocytes. Incubation with AEA or 2-AG (at ∼0.3 µ*M* for 4 h) had differential effects on the expression of ECS components: AEA increased *CNR1*, whereas 2-AG increased the expression of *CNR2* and *FAAH* and tended to increase *GPR55* and *NAPEPLD*. In contrast to our hypothesis, incubation with either AEA or 2-AG did not affect the level of expression of inflammatory genes in the leukocytes either with or without LPS stimulation. The immune system is mostly regulated by the ECS via *CNR2* activation (Basu and Dittel, 2011; Zachut et al., 2025), but growing evidence indicates the involvement of *CNR1* and other eCB receptors, such as *PPARG* and *GPR55* (Malek et al., 2015; Heming et al., 2018). Malek et al. (2015) incubated rat primary microglial cultures with AEA (1.0 µ*M* for 15 min), followed by LPS stimulation (100 ng/mL for 24 h), and found that LPS increased the expression of *CNR2* in the cells that were pretreated with CB2 antagonist (AM-630; 0.5 µ*M*) but decreased the expression of *CNR1* regardless of treatment with AEA, CB1 antagonist (AM-251; 0.25 µ*M*), CB2 antagonist (AM-630; 0.5 µ*M*), or GPR18/55 antagonist (CID-16020046; 0.25 µ*M*). In contrast, LPS stimulation had no major effects on *CNR1* expression in rodent macrophages (Carlisle et al., 2002) or in human peripheral lymphocytes (Maccarrone et al., 2001). These studies support our findings that LPS had no major effect in *CNR1* gene expression compared with nonstimulated leukocytes. In rodent macrophages and macrophage-like cells, *CNR2* mRNA and protein levels were downregulated by exposure to LPS, with or without priming with interferon gamma, and the overall levels of *CNR2* expression were dependent on the cells' immunological activation status (Carlisle et al., 2002). Similarly, we found that CTL+LPS reduced the expression of *CNR2* compared with the non-LPS-challenged cells (CTL, AEA, or 2-AG), and versus cells pre-incubated with eCB followed by LPS challenge (AEA+LPS or 2AG+LPS). In vivo, in nonpregnant Simmental cows (>120 DIM) plasma concentration of AEA was ∼0.26 n*M* and 2-AG was ∼3.60 n*M* (van Ackern et al., 2021). In vitro studies have tested the effects of a range of eCB concentrations in different cell types and species. In human mononuclear cells, Berdyshev et al. (1997) reported that incubation with AEA at 0.3 to 3 µ*M* for 30 min inhibited cytokine production. In bovine endothelial cells, Walker et al. (2022) found that incubation with AEA at 0.5 µ*M* for 4 h induced an oxidative stress response. Gasperi et al. (2014) showed a dose-dependent effect of 2-AG at 0.1 to 10 µ*M* on molecules involved in human endothelial cell/leukocyte interactions. Together, our selected eCB concentrations fall within the range reported in those studies, and the effects of eCB on leukocytes likely depend on cell type and state, the type of inflammatory stimulation, eCB concentrations, and time of incubation.

In our study, LPS reduced the expression of 3 eCB receptors, but only the main receptors *CNR1* and *CNR2* were modulated by incubation with their native ligands. These findings suggest that their activation is highly adaptable, contributing to maintenance of immune homeostasis. We infer that the observed downregulation of the expression of eCB receptors by LPS could be a strategy to regulate the inflammatory response. It has been suggested that LPS-induced downregulation of *PPARG* is transient and may be a part of early acute inflammatory response (Welch et al., 2003; Heming et al., 2018). Therefore, it would be interesting to explore the interplay between inflammatory response and ECS in later stages of inflammation. We found that LPS differentially modulates the expression patterns of the key metabolic enzymes for both AEA and 2-AG, suggesting a shift toward enhanced 2-AG catabolism and perhaps also toward increase in AEA biosynthesis. Moreover, compared with CTL, ex vivo supplementation with 2-AG tended to upregulate the gene expression of the eCB synthesizing enzyme *NAPEPLD* and upregulated the degradation enzyme *FAAH*, both of which are involved in AEA metabolism. In immune cells, 2-AG is a full agonist of both CB1 and CB2, whereas AEA acts as a partial agonist (Basu and Dittel, 2011). It has been shown that 2-AG induces leukocytes migration and adhesion to vascular endothelial cells and extracellular matrix proteins via a CB2-dependent mechanism, which may contribute to the recruitment of immune cells to the inflammation site (Gokoh et al., 2005). To date, no direct evidence has connected 2-AG with regulation of the *FAAH* or *NAPEPLD* enzymes in immune cells. Conversely, FAAH may play a minor role in hydrolyzing 2-AG (Rouzer and Marnett, 2011). In human peripheral lymphocytes incubated with LPS (100 µg/mL for 1 h) and CB1 (SR141716) or CB2 (SR144528) antagonists, at 1 µ*M* for 24 h, LPS did not affect the transcription levels or the binding capacity of the cannabinoid receptors, but reduced *FAAH* expression, thereby inhibiting its activity levels concomitantly with AEA levels elevation; thus, LPS may indirectly affect the receptors' functionality (Maccarrone et al., 2001). Further studies are required to elucidate the effects that inflammatory stimulus exerts on CB1 and CB2 receptors in a context of local eCB concentrations and their effects on the inflammatory response.

In dairy cows, downregulation of the ECS by dietary means in vivo has been associated with changes in immune function during the transition period (Kra et al., 2022, 2023; Zachut et al., 2025). In the current ex vivo study, LPS had a strong effect on several components of the ECS in leukocytes, which could be a part of the regulation of the inflammatory process. Although we did not observe effects of eCB on the examined cytokines' transcriptions, the regulation of inflammation may occur at post-transcription level or receptor functionality. Alternately, it could be postulated that the effects could be related to the stage of lactation (postpartum vs. midlactation). In conclusion, this is the first reductionist step in the exploration of the regulation of immune cells by the ECS in dairy cows. The complexity of immune regulation and ECS activation in leukocytes requires further studies to fully elucidate the mechanistic effects underlying these responses.
